# Candidalysin Crucially Contributes to Nlrp3 Inflammasome Activation by Candida albicans Hyphae

**DOI:** 10.1128/mBio.02221-18

**Published:** 2019-01-08

**Authors:** Ona Rogiers, Ulrika C. Frising, Soňa Kucharíková, Mary Ann Jabra-Rizk, Geert van Loo, Patrick Van Dijck, Andy Wullaert

**Affiliations:** a VIB-KU Leuven Center for Microbiology, Leuven, Belgium; b KU Leuven Laboratory of Molecular Cell Biology, Institute of Botany and Microbiology, Leuven, Belgium; c Department of Internal Medicine and Pediatrics, Ghent University, Ghent, Belgium; d VIB-UGent Center for Inflammation Research, VIB, Ghent, Belgium; e Department of Microbiology and Immunology, School of Medicine, University of Maryland, Baltimore, Maryland, USA; f Department of Oncology and Diagnostic Sciences, School of Dentistry, University of Maryland, Baltimore, Maryland, USA; g Department of Biomedical Molecular Biology, Ghent University, Ghent, Belgium; h Ghent Gut Inflammation Group (GGIG), Ghent University, Ghent, Belgium; University of Texas Health Science Center

**Keywords:** *Candida albicans*, NLRP3, candidalysin, inflammasome, primary macrophages

## Abstract

Candida albicans is an opportunistic fungal pathogen that can cause life-threatening infections, particularly in immunocompromised patients. C. albicans induced activation of the Nlrp3 inflammasome, leading to secretion of bioactive interleukin 1β (IL-1β) is a crucial myeloid cell immune response needed for antifungal host defense. Being a pleiomorphic fungus, C. albicans can provoke Nlrp3 inflammasome responses only upon morphological transformation to its hyphal appearance. However, the specific hyphal factors that enable C. albicans to activate the Nlrp3 inflammasome in primary macrophages remain to be revealed. Here, we identify candidalysin, a peptide derived from the hypha-specific *ECE1* gene, as a fungal trigger for Nlrp3 inflammasome-mediated maturation and secretion of IL-1β from primary macrophages. Direct peptide administration experiments showed that candidalysin was sufficient for inducing secretion of mature IL-1β from macrophages in an Nlrp3 inflammasome-dependent manner. Conversely, infection experiments using candidalysin-deficient C. albicans showed that candidalysin crucially contributed to the capacity of this fungus to induce maturation and secretion of IL-1β from primary macrophages. These complementary observations identify the expression of candidalysin as one of the molecular mechanisms by which hyphal transformation equips C. albicans with its proinflammatory capacity to elicit the release of bioactive IL-1β from macrophages.

## Abstract (precis)

Candidiasis is a potentially lethal condition that is caused by systemic dissemination of Candida albicans, a common fungal commensal residing mostly on mucosal surfaces. The transition of C. albicans from an innocuous commensal to an opportunistic pathogen goes hand in hand with its morphological transformation from a fungus to a hyphal appearance. On the one hand, the latter manifestation enables C. albicans to penetrate tissues, while on the other hand, the expression of many hypha-specific genes also endows it with the capacity to trigger particular cytokine responses. The Nlrp3 inflammasome is a crucial component of the innate immune system that provokes release of the IL-1β cytokine from myeloid cells upon encountering C. albicans hyphae. Our study reveals the peptide candidalysin as one of the hypha-derived drivers of Nlrp3 inflammasome responses in primary macrophages and, thus, contributes to better understanding the fungal mechanisms that determine the pathogenicity of C. albicans.

## OBSERVATION

Candida albicans is a commensal fungus that can transform to a highly pathogenic organism capable of establishing severe mycoses in immunocompromised patients (1, 2). Many factors discriminating between the harmless versus potentially damaging states of C. albicans relate to its appearance as a pleiomorphic fungus. Although its yeast-to-hypha morphological transition boosts the expression levels of many C. albicans proteins, such as adhesins and secreted enzymes (3–6), the individual impacts of these hypha-specific fungal factors on host innate immunity are not always clear.

**FIG 1:**
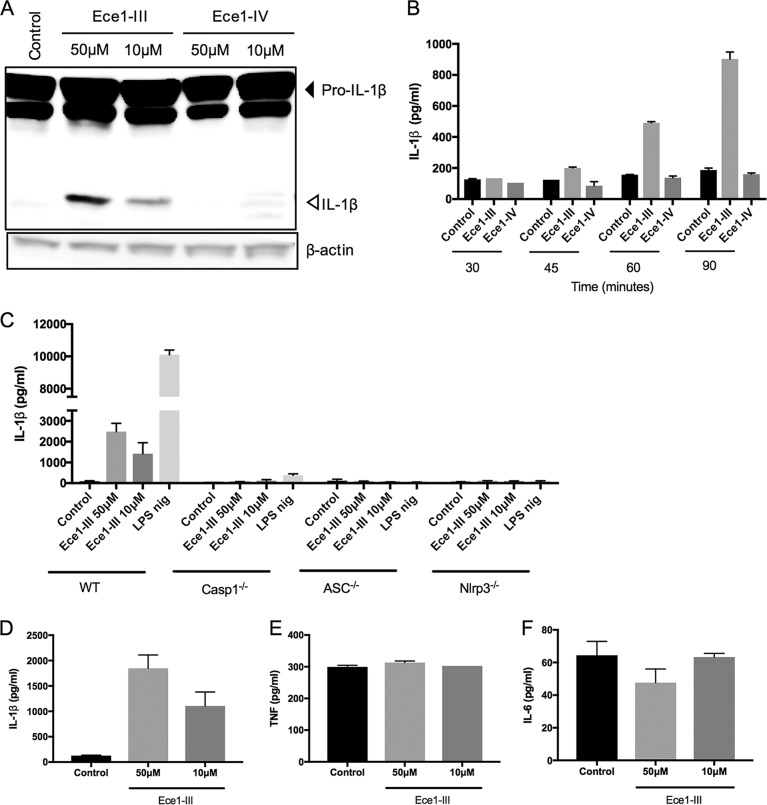
Candidalysin induces Nlrp3 inflammasome-mediated IL-1β secretion and maturation in primary macrophages. (A) Wild-type BMDMs were primed with 100 µg/ml curdlan for 3 h and then left untreated (control) or incubated with the indicated concentrations of either the Ece1-III or the Ece1-IV peptide. At 2 h posttreatment, cell lysates were immunoblotted for IL-1β maturation. (B) Wild-type BMDMs were primed with 100 µg/ml curdlan for 3 h and then left untreated (control) or incubated with 50 µM Ece1-III peptide. Supernatants were collected at the indicated time points after peptide administration and analyzed for secreted IL-1β by multiplex Luminex. (C) BMDMs of the indicated genotypes were primed with 100 µg/ml curdlan for 3 h and then left untreated (control) or were incubated with the indicated concentrations of Ece1-III. At 90 min posttreatment, culture supernatants were analyzed for secreted IL-1β by the multiplex Luminex assay. As a positive control, BMDMs were primed with LPS (500 ng/ml) for 3 h and incubated with nigericin (nig) for 45 min. (D to F) Wild-type BMDMs were primed with 100 µg/ml curdlan for 3 h, after which the culture medium was aspirated and replaced with either control medium or medium containing the indicated concentrations of Ece1-III. At 90 min posttreatment, the culture supernatants were analyzed for secreted IL-1β (D), TNF (E), and IL-6 (F) by the multiplex Luminex assay. Data shown in panels B to F are the means ± standard deviations (SD) of results from triplicate wells from a representative experiment out of two independent experiments. Data shown in panel A are representative of two independent experiments.

The Nlrp3 inflammasome is a signaling complex that mediates maturation and release of the proinflammatory cytokine interleukin 1β (IL-1β), crucial for protecting the host against systemic C. albicans infection (7–10). While several studies showed that C. albicans transition from yeast cells to hyphae was necessary to activate Nlrp3 inflammasomes, additional hypha-derived factors are needed to activate Nlrp3 (10–13). Based on reports that several bacteria utilize toxins forming pores in the cellular membrane to cause the efflux of intracellular K^+^ for triggering Nlrp3-driven inflammasome activation (14), we hypothesized that the cellular membrane-damaging potential of the *Ece1III* toxin, a C. albicans peptide also termed candidalysin that is encoded by the hypha-specific *ECE1* gene (15), might initiate inflammasome responses. To investigate this possibility, we performed experiments in which we mimicked fungal β-glucan-mediated inflammasome priming by treating primary bone marrow-derived macrophages (BMDMs) with curdlan and then administered *Ece1III* peptide to these curdlan-primed cells (see the supplemental material). Interestingly, while the *ECE1*-derived control *Ece1IV* peptide did not induce IL-1β processing, the *Ece1III* peptide had a dose-dependent capacity to induce IL-1β maturation in curdlan-primed macrophages (Fig. 1A), leading to increased secretion of IL-1β, starting from 60 min after stimulation (Fig. 1B). In addition, the *Ece1III* peptide induced IL-1β secretion from wild-type (WT) macrophages but not from macrophages lacking either caspase-1, ASC, or Nlrp3 (Fig. 1C). While these findings identify candidalysin as a canonical Nlrp3 inflammasome activator, we next verified the specificity of this peptide-induced cytokine response. Because curdlan by itself provokes secretion of NF-κB-dependent cytokines, we performed experiments in which we washed away this priming agent prior to candidalysin treatment for specifically assessing cytokine induction by the latter. These experiments demonstrated that candidalysin-treated cells specifically secreted IL-1β without releasing the inflammasome-independent cytokines tumor necrosis factor (TNF) and IL-6 (Fig. 1D to F). Together, these results showed that candidalysin was sufficient to specifically provoke secretion of IL-1β from primary macrophages by activating the Nlrp3 inflammasome.

**FIG 2:**
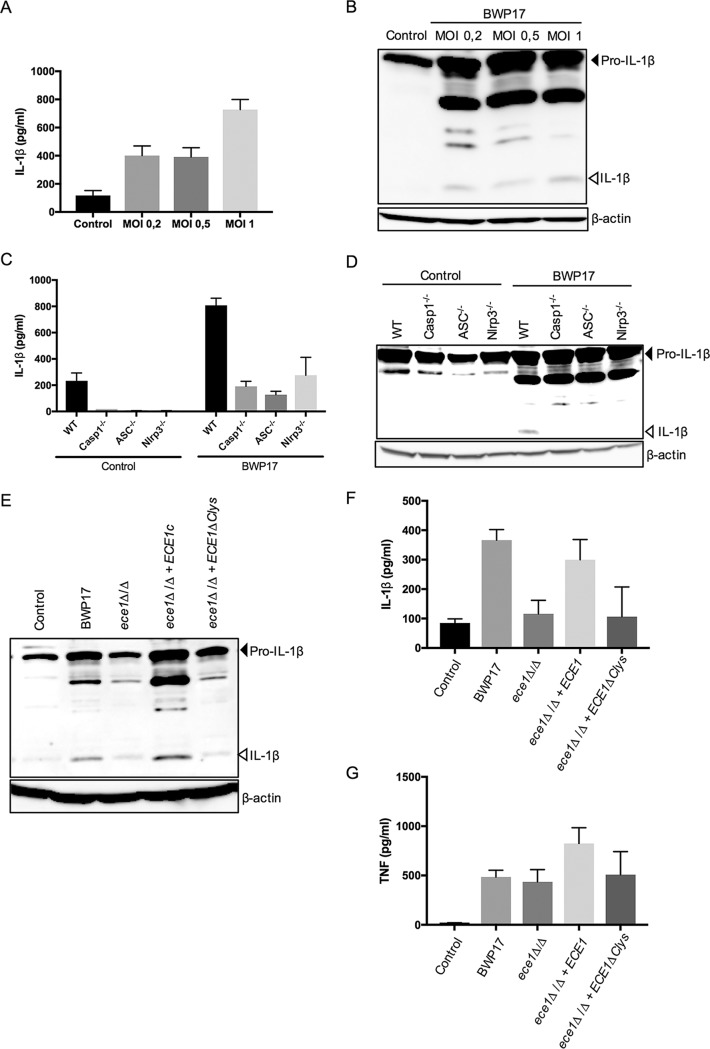
Candidalysin crucially contributes to C. albicans-induced IL-1β secretion and maturation in unprimed primary macrophages. (A, B) Naïve wild-type BMDMs were left untreated (control) or were incubated at the indicated MOIs of live C. albicans. At 24 h postinfection, culture supernatants were analyzed for secreted IL-1β by enzyme-linked immunosorbent assay (ELISA) (A) and cell lysates were immunoblotted for IL-1β maturation (B). (C, D) Naïve BMDMs of the indicated genotypes were left untreated (control) or were incubated at an MOI of 0.5 with live C. albicans cells. At 24 h postinfection, culture supernatants were analyzed for secreted IL-1β by ELISA (C) and cell lysates were immunoblotted for IL-1β maturation (D). (E to G) Naïve wild-type BMDMs were left untreated (control) or were incubated at an MOI of 0.5 with the indicated C. albicans strains. At 24 h postinfection, cell lysates were immunoblotted for IL-1β maturation (E) and culture supernatants were analyzed for secreted IL-1β (F) and TNF (G) by the Luminex assay. Data shown in panels A, C, F, and G are the means ± SD of results from triplicate wells from a representative experiment out of two independent experiments. Data shown in panels B, D, and E are representative for two independent experiments.

Our next experiments aimed to address the endogenous fungal contribution of candidalysin to Nlrp3 activation in primary macrophages. In accordance with a prior report showing that C. albicans by itself can perform the priming as well as the activation step for eliciting Nlrp3 inflammasome responses from naive BMDMs (10), incubating unprimed macrophages with C. albicans at a multiplicity of infection (MOI) of 0.2 for 24 h sufficed to provoke IL-1β secretion (Fig. 2A). Moreover, Western blotting showed that infecting naive macrophages with these low numbers of C. albicans was sufficient for detecting IL-1β maturation (Fig. 2B). In contrast to WT BMDMs, macrophages lacking either caspase-1 or ASC failed to mature or secrete IL-1β upon C. albicans infection (Fig. 2C and D). Upstream of ASC and caspase-1, both Nlrp3 and Nlrc4 have been suggested as inflammasome receptors upon C. albicans infection in mice (8, 10, 16–18). However, deleting Nlrp3 fully abrogated C. albicans-induced IL-1β maturation and secretion to the same extent as deleting caspase-1 or ASC did (Fig. 1C and D). These observations showed that overnight infection of unprimed BMDMs with C. albicans specifically activated the Nlrp3 inflammasome and, thus, validated this experimental setting for assessing the physiological contribution of candidalysin to whole-fungus-induced Nlrp3 inflammasome responses. For this purpose, we used the parental wild-type BWP17 strain and an *ece1*Δ/Δ strain that lacks the entire *ECE1* gene product, from which candidalysin is produced (see the supplemental material). In addition, we also used *ece1*Δ/Δ strains that were reconstituted either with the complete *ECE1* gene (*ece1*Δ/Δ+ECE1) or with an *ECE1* gene from which the candidalysin-encoding sequence was deleted (*ece1*Δ/Δ+ECE1ΔClys). While WT C. albicans induced both maturation and secretion of IL-1β in unprimed macrophages, both of these inflammasome responses were diminished upon infection with the *ece1*Δ/Δ strain, demonstrating that an *ECE1*-derived fungal factor was crucial for C. albicans-induced Nlrp3 inflammasome activation (Fig. 2E and F). As expected, reconstituting the *ece1*Δ/Δ strain with the complete *ECE1* gene restored its capacity to induce IL-1β maturation and secretion to levels similar those of WT fungi (Fig. 2E and F). In contrast, reconstituting the *ece1*Δ/Δ strain with a candidalysin-deficient *ECE1* gene did not elicit higher IL-1β secretion levels from macrophages than those elicited by the *ece1*Δ/Δ strain (Fig. 2E). In addition, the inability of inducing IL-1β maturation by the *ece1*Δ/Δ strain could not be corrected by expressing a candidalysin-deficient *ECE1* gene (Fig. 2F). In contrast to what occurred with IL-1β, levels of release of the inflammasome-independent cytokine TNF did not differ when infections with the various C. albicans strains were compared (Fig. 2G), showing that candidalysin specifically controlled inflammasome-dependent cytokine secretion.

In summary, we showed that the Nlrp3 inflammasome-activating potential of C. albicans at least partially relies on candidalysin. In fact, it was known that candidalysin administration provokes IL-1β release in human TR146 epithelial cells (19). However, while it is not clear whether inflammasome activation takes part in this epithelial candidalysin effect, we show that IL-1β secretion upon candidalysin administration to macrophages depends entirely on the Nlrp3 inflammasome. As various bacterial virulence factors activate the Nlrp3 inflammasome due to pore-forming capacities (14), it is conceivable that the Nlrp3 inflammasome-activating potential of candidalysin derives from an ability to damage cellular membranes. Indeed, a recent study showed that candidalysin-induced Nlrp3 inflammasome activation in macrophages was associated with membrane permeabilization and decreased cytosolic K^+^ levels (20). While this study, thus, confirmed our observations and identified K^+^ efflux as the candidalysin-induced mechanism triggering Nlrp3 inflammasome activation, it is striking that a candidalysin-deficient C. albicans strain used in this study was not defective in IL-1β secretion at 5 h postinfection in lipopolysaccharide (LPS)-primed murine macrophages (20). This seems in contrast with our observation showing that naive BMDMs infected with the *ece1*Δ/Δ+ECE1ΔClys strain for 24 h displayed less IL-1β maturation and secretion. However, as the *ece1*Δ/Δ+ECE1ΔClys strain still provoked residual amounts of IL-1β processing and secretion from unprimed macrophages, both our experiments and those of Kasper et al. (20) indicate that C. albicans harbors multiple redundant factors capable of activating the Nlrp3 inflammasome. In this respect, recent genome-wide screening studies performed with immortalized macrophages identified a myriad of fungal factors as potential contributors to C. albicans-induced inflammasome activation (21, 22). Given the discrepancy between the crucial contributing role for candidalysin in naive macrophages observed in our study versus its dispensable role in LPS-primed macrophages used in the Kasper et al. study (20), it is conceivable that the various C. albicans Nlrp3 activators act with different kinetics and that their activities depend on specific host cell factors. Along these lines, it is possible that activated murine BMDMs are prone to rapid candidalysin-independent inflammasome activation, while naive macrophages may undergo a slower candidalysin-dependent Nlrp3 inflammasome activation. In conclusion, while additional mechanisms certainly exist, we identified candidalysin as a hyphal C. albicans factor that crucially contributes to Nlrp3 inflammasome activation in naive murine macrophages.

10.1128/mBio.02221-18.1 TEXT S1 Supplemental materials and methods. Download Text S1, DOCX file, 0.05 MB. Copyright © 2019 Rogiers et al. 2019 Rogiers et al. This content is distributed under the terms of the Creative Commons Attribution 4.0 International license.

10.1128/mBio.02221-18.2 TABLE S1 C. albicans strains used in this study. Download Table S1, DOCX file, 0.01 MB. Copyright © 2019 Rogiers et al. 2019 Rogiers et al. This content is distributed under the terms of the Creative Commons Attribution 4.0 International license.
