# Supplementary material for: Candidalysin Crucially Contributes to Nlrp3 Inflammasome Activation by Candida albicans Hyphae
Source: mBio. 2019 Jan 8;10(1):e02221-18. doi: 10.1128/mBio.02221-18 (PMC6325245; doi:10.1128/mBio.02221-18)
Supplement: TEXT S1 [file mBio.02221-18-s0001.docx]

**Candidalysin crucially contributes to Nlrp3 inflammasome activation by *Candida albicans* hyphae**

Ona Rogiers^a,b,c,d^, Ulrika C. Frising^c,d^, Soňa Kucharíková^a,b^, Mary Ann Jabra-Rizk^e^, Geert van Loo^d,f^, Patrick Van Dijck^a,b,#^ and Andy Wullaert^c,d,f,g,#^

**Text Supplementary 1**

**MATERIALS AND METHODS**

**Animals**

C57BL/6J mice were originally obtained from Charles River laboratories and bred in-house. Caspase-1^-/-^(1), ASC^-/-^(2) and Nlrp3^-/-^(3) mice have been described. All mice used in this study were generated on C57BL/6J background or were backcrossed at least 10 generations to C57BL/6J background and subsequently bred in-house in individually ventilated cages under specific pathogen-free conditions. All animal experiments were performed according to institutionally approved protocols according to national (Belgian Laws 14/08/1986 and 22/12/2003, Belgian Royal Decree 06/04/2010) and European (EU Directives 2010/63/EU, 86/609/EEG) animal regulations.

**Primary Macrophage Differentiation and Stimulation**

Murine primary bone marrow derived macrophages (BMDMs) were obtained after isolating mouse bone marrow cells from femurs and tibias. Subsequently, bone marrow cells were differentiated for six days at 37°C and 5% CO_2_ in Iscove’s Modified Dulbecco’s Medium (IMDM) supplemented with 30% L929 cell conditioned medium, 10% heat-inactivated fetal bovine serum (FBS), 1% non-essential amino acids and 1% penicillin-streptomycin. BMDMs were then seeded in 24-well plates and were used the following day for experiments. For *C. albicans* infections, unprimed cells were incubated with live *C. albicans,* overnight culture diluted to the indicated multiplicity of infection (MOI), for 24 hours. For peptide stimulation experiments, cells were either left untreated or primed with 100 µg/ml Curdlan (Invivogen) for 3 hours prior to treatment with Ece1-III (candidalysin, Ac-SIIGIIMGILGNIPQVIQIIMSIVKAFKGNK, Caslo) or Ece1-IV (control, Ac-MKFSKIACATVFALSSQAAIIHHAPEFNMK, Caslo) peptides for 2 hours.

**Strains and Media**

All *C. albicans* strains were streaked on YPD (1% yeast extract, 2% peptone, 2% glucose) agar plates and incubated at 37 °C. Overnight culture of *C. albicans* strains were prepared in liquid YPD medium at 30 °C. The parental strain (BWP17+CIp30) and Ece1 deletion mutants have been described (4) and are listed in Table 1. All strains were kindly provided by Prof. Bernhard Hube (Leibniz Institute for Infection Biology, Hans Knöll Institute, Jena, Germany).

**Western Blotting**

Cells and culture supernatants were incubated in cell lysis buffer (20mM Tris HCl (pH 7.4), 200 mM NaCl, and 1%NP-40) for 10 minutes on ice, followed by denaturing in Laemmli buffer at 95°C for 10 minutes. Cell lysates were separated by 12%, 16% or 8% SDS-PAGE prior to transfer to polyvinylidene fluoride (PVDF) or nitrocellulose membranes using a trans blot turbo kit (170-4273, Biorad). Membrane blocking, antibody incubation and washing was performed in PBS supplemented with 0.05% (v/v) Tween 20 and 3% (w/v) skimmed dry milk. Blots were incubated overnight at 4^o^C with primary antibodies against IL-1β (GTX74034, 1:3000, GeneTex) and β-actin (sc-47778-HRP, Santa Cruz Biotechnology). Horseradish peroxidase-conjugated anti-rabbit (111-035-144, 1:5000, Jackson ImmunoResearch Laboratories) antibody was used to detect proteins by enhanced chemiluminescence (Thermo Scientific).

**Cytokine Analysis**

Cytokine levels in cell culture supernatants were determined by magnetic bead-based multiplex assay using Luminex technology (Bio-Rad) or by ELISA (DY401-05, R&D systems) according to the manufacturer’s instructions.

**References**

1. Van Gorp H, Saavedra PH, de Vasconcelos NM, Van Opdenbosch N, Vande Walle L, Matusiak M, Prencipe G, Insalaco A, Van Hauwermeiren F, Demon D, Bogaert DJ, Dullaers M, De Baere E, Hochepied T, Dehoorne J, Vermaelen KY, Haerynck F, De Benedetti F, Lamkanfi M. 2016. Familial Mediterranean fever mutations lift the obligatory requirement for microtubules in Pyrin inflammasome activation. Proc Natl Acad Sci U S A 113:14384-14389.

2. Mariathasan S, Newton K, Monack DM, Vucic D, French DM, Lee WP, Roose-Girma M, Erickson S, Dixit VM. 2004. Differential activation of the inflammasome by caspase-1 adaptors ASC and Ipaf. Nature 430:213-8.

3. Kanneganti TD, Ozoren N, Body-Malapel M, Amer A, Park JH, Franchi L, Whitfield J, Barchet W, Colonna M, Vandenabeele P, Bertin J, Coyle A, Grant EP, Akira S, Nunez G. 2006. Bacterial RNA and small antiviral compounds activate caspase-1 through cryopyrin/Nalp3. Nature 440:233-6.

4. Moyes DL, Wilson D, Richardson JP, Mogavero S, Tang SX, Wernecke J, Hofs S, Gratacap RL, Robbins J, Runglall M, Murciano C, Blagojevic M, Thavaraj S, Forster TM, Hebecker B, Kasper L, Vizcay G, Iancu SI, Kichik N, Hader A, Kurzai O, Luo T, Kruger T, Kniemeyer O, Cota E, Bader O, Wheeler RT, Gutsmann T, Hube B, Naglik JR. 2016. Candidalysin is a fungal peptide toxin critical for mucosal infection. Nature 532:64-8.
