# Supplementary material for: Candidalysin Crucially Contributes to Nlrp3 Inflammasome Activation by Candida albicans Hyphae
Source: mBio. 2019 Jan 8;10(1):e02221-18. doi: 10.1128/mBio.02221-18 (PMC6325245; doi:10.1128/mBio.02221-18)
Supplement: TABLE S1 [file mBio.02221-18-st001.docx]

**Table S1. *C. albicans* strains used in this study**

| **Strain name** | **Strain/Gene function** | **Reference** |
| --- | --- | --- |
| BWP17 (+ CIp30) | Parental strain | **(4)** |
| *ece1*Δ/Δ | Hyphae associated protein | **(4)** |
| *ece1*Δ/Δ + *ECE1* | Reconstituted strain | **(4)** |
| *ece1*Δ/Δ + *ECE1*ΔClys | Partially reconstituted | **(4)** |
